# Cyclic Behavior of Gabled Frames with Web-Tapered Columns and Rafters

**DOI:** 10.3390/ma16010307

**Published:** 2022-12-28

**Authors:** Haisheng Yang, Mingzhou Su, Yong Xiao, Dan Gan

**Affiliations:** 1 Chongqing Steel Structure Industry Co., Ltd., Chongqing 400080, China; 2 School of Civil Engineering, Xi’an University of Architecture and Technology, Xi’an 710055, China; 3 College of Civil Engineering, Chongqing Jiaotong University, Chongqing 400074, China; 4 State Key Laboratory of Mountain Bridge and Tunnel Engineering, Chongqing 400074, China; 5 School of Civil Engineering, Chongqing University, Chongqing 400045, China

**Keywords:** steel gabled frames, end-plate joint, cyclic load, failure mode, seismic performance, slender section, tapered section, finite element analysis

## Abstract

Cyclic loading tests were conducted on three 1/2-scale, half-bay steel gabled frames (SGFs) to investigate their seismic performance. The three specimens with reduced joint stiffness were designed based on the prototype drawing shown in China design guideline 02SG518—1: specimen SV1 with a reduced thickness of the joint end-plate and bolt diameter, specimen SV2 with a reduced number of bolts, and specimen SV3 with a reduced bolt diameter. The load capacity, rotational stiffness, rotational capacity, and ultimate failure mode of specimens SV1, SV2, and SV3 were investigated. The experimental results showed that specimen SV1 failed due to the local buckling of the lower flange of the rafter, and specimens SV2 and SV3 due to the local buckling of upper flange of the rafter. The joint zone of all specimens kept well, indicating that the prototype joint had a large margin of safety. The hysteresis curves of all specimens were not full, and the ductility and energy dissipation capacity were limited. The end-plate thickness, bolt diameter, and steel grade affected the hysteresis performance of the SGF little. A refined finite element model was established, and the predicted results compared well with the test results. The test and analysis results demonstrated that there was slight utilization and distribution of post-buckling strength.

## 1. Introduction

**Figure 1:**
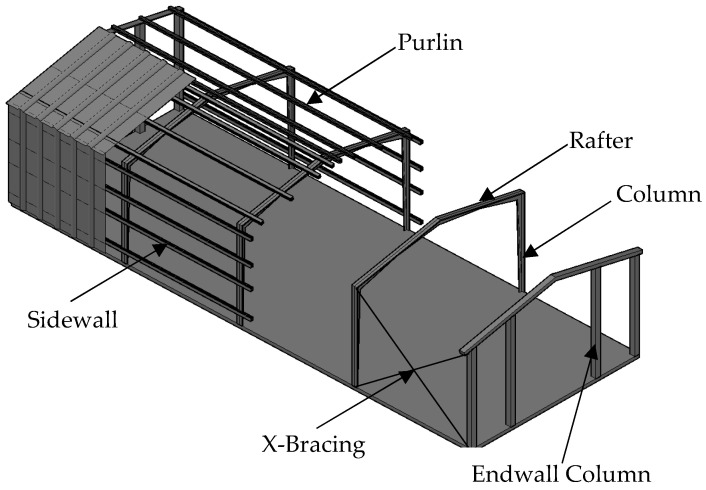
Steel-gabled frames.

Steel gabled frames (SGFs) are structural systems consisting of rafters, columns, purlins, endwall column, roofs, and X-Bracings mainly, as shown in Figure 1. The main load-carrying members of SGFs are columns and rafters, which usually adopt H-section members with web-tapered cross-sections. The web-tapered cross-sections are usually optimized according to the bending moment to reduce steel usage and minimize construction costs. The variation of web-depth depends on the distribution of strong-axial moments along the length of the member, so SGFs mostly have a tapered web along the whole length of the column and part of the length of the rafter. In addition, the simple connection between the column and the foundation of SGFs is usually adopted with the aim of avoiding the transfer of bending moments to the foundation, and making the connection details simpler and the design lighter [1].

With the advancement of design theory, construction technology, and building materials, SGF systems have been widely used in low-rise non-residential buildings worldwide because of the following advantages:

1. In terms of structural weight and material consumables, the self-weight of SGFs is only 1/10 to 1/8 of the reinforced concrete structure. In addition, the steel consumption of SGFs is 10–20% less than that of steel frames because of allowing the use of web post-buckling strength of members with a non-compact or slender section.
2. Fast erection of SGFs is possible due to the use of bolted connections during construction. Even though SGFs have a large members size, the light weight makes them convenient to transport.
3. Long spans and large space without middle columns can be achieved, and the large space provides convenience for layout.
4. Technically, non-compact and slender tapered-web members are becoming popular with the application of building information modeling and automated robotic welding technology [2], which makes the manufacturing of tapered members from steel plates efficient.

In general, significant economic advantages of SGFs can be achieved because of the fewer steel consumptions, large internal space, efficient transportation, and improved connection technology have led to. The maximum design span of SGFs specified in Technical Code for Steel Structure of Light-Weight Buildings with Gabled Frames GB51022-2015 [3] is 48 m, and it should be noted that the maximum span of SGFs built in China has reached 72 m. Thus, SGFs are extensively used in aircraft hangars, industrial plants, warehouses, stadiums, conference halls, swimming pools, and other places that require large spaces [4], indicating great application potential.

For steel frames, selected structural elements of the beam-to-column joints are used to dissipate energy when subjected to strong earthquakes. By replacing these elements which are usually referred to as fuse elements, the structural system can quickly resume their service. For example, He et al. [5] developed a steel connection composed of steel angles. These steel angles were designed as fuse elements for energy dissipation and post-earthquake replacement. He et al. [6,7,8] proposed a connection system compatible with concrete slabs by restraining damage to the fuse elements at the bottom flange, and the energy dissipation capability of the connection system has been experimentally demonstrated to be stable and full. Chen et al. [9] carried out an experimental study of the performance of the joint zone of welded thin-walled steel beams and columns under large plastic strain cyclic loading; a microscopically based ductile fracture model was proposed and the ductile cracking process in the joint zone after buckling was numerically simulated; and parametric analyses were performed to investigate the effects of equivalent joint zone width–thickness ratio, axial load ratio and initial geometric defects on the seismic performance, crack generation, and expansion of the connection. Pan et al. [10] experimentally investigated the effect of vertically placed end-plate on the stability of steel beam–column connection region. It was found that the vertical connection plate can effectively reduce the shear buckling in the panel zone, and an improved method to evaluate the shear stability in the panel region by equivalent width–thickness ratio was proposed. Wang et al. [11] studied the potential of cast steel technology in earthquake engineering and proposed two new seismic cast modular joints for H-beam-thin-walled square steel pipe columns; the tensile and cyclic tests showed that the cast modular joints had excellent seismic performance in terms of good ductility and energy dissipation capacity.

For structural members of SGFs, several experimental and analytical studies were performed by Lee et al. [12,13,14], which provided design guidance and provisions for the design of the web-tapered members. Then, the web-tapered members were further investigated by Forest and Murray [15], Shiomi and Kurata [16], Ashakul and Murray [17], and Mohamed and Ibrahim [18].

As for the seismic performance of SGF systems, Xu et al. [19] examined the failure mode of SGFs with tapered members under vertical and cyclic horizontal loads. The experimental results showed that the final failure of the frame was characterized by local buckling, which located at the rafter section close to the rafter–column joint and at the rafter–rafter connection. Hong and Uang [20] conducted seismic performance tests on full-size SGFs with non-compact tapered-web members. The test results showed that the SGFs failed due to lateral-torsional buckling of the web-tapered rafters. Similar conclusion was reported from an experiment investigation conducted by Wang et al. [21]. Hwang et al. [22] carried out shake table tests on 1/5 scale SGFs and the experimental results showed that premature local buckling occurred before lateral-torsional buckling in cases the width-to-thickness and depth-to-thickness ratios did not satisfy the specified requirements of compact sections. Smith [23] performed two shake tests on full-scale SGFs: one specimen was with light-metal-plate sidewalls and the other with heavy-concrete sidewalls; the results showed that all the four rafters exhibited lateral-torsional buckling near the pinch point at the failure stage. Su et al. [24] conducted 1/3 scale shaking table tests of SGFs; the test results showed that the failure modes were bolts loosening at the column bases and local buckling of the rafters accompanied by lateral torsional buckling; additionally, the obvious stiffness degradation in the elastic-plastic stage indicated that the ductility of lightweight SGFs was limited. Mohammad et al. [4] analyzed the seismic reliability of SGFs at design basis earthquake (DBE) and maximum considered earthquake (MCE). On this basis, the hazard curves of the study region were combined with the fragility curves to evaluate the seismic reliability of the structure at DBE and MCE hazard levels, and the annual exceedance rate of the structure was derived. The seismic reliability results showed that the existing seismic design guidelines lead to overly conservative design of SGFs, especially when they have long periods, with a safety margin of about 58% for SGFs with long-period at the MCE hazard level.

The above studies investigated the seismic performance of SGFs with non-compact and slender tapered-webs and the performance of tapered components. The beam–column joints are crucial for force transferring, and affect or even dominate the seismic performance of the whole structure [25]. The design method of SGF joints in GB51022-2015 [3] was referred to the design of steel roof frame in GB 50011 [26], which was mainly based on the conception or the experience. Yet, SGFs are obviously different from steel roof frames. Hence, this investigation assessed the performance of three joints designed by GB51022-2015 [3] through tests to provide a basis for evaluating the seismic performance of joints of SGFs. Based on the 18-6c Rigid frame in GB51022-2015 [3], the capacity and stiffness of the joints were weakened by three methods: reducing the end-plate thickness, the number of bolts in the joint zone, and the strength grade of the bolts, respectively. Therefore, three specimens with weakened joint zones were experimentally and numerically investigated to evaluate whether the seismic performance of the 18-6c rigid frame could satisfy the seismic design requirements. By analyzing the seismic performance of three specimens under hysteretic loading, design methods in GB51022-2015 [3] were estimated. In addition, the experimental results and finite element results were used to investigate whether the post-buckling strength of slender tapered sections in SGFs could be utilized.

## 2. Experimental Program

### 2.1. Experimental Design

**Figure 2:**
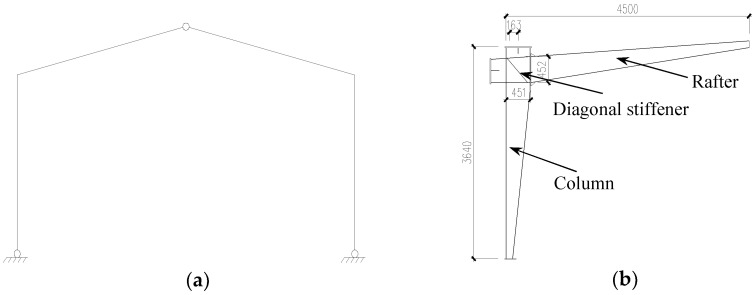
Gabled frame model. (**a**) Three-hinged gabled frame; (**b**) ½-bay gabled frame.

The 1/2-bay gabled frame model was used to simulate the three-hinged gabled frame subjected to cyclically lateral loading, as shown in Figure 2; this experimental method was feasible and economical. For all specimens, the end-plates of joint zone were placed vertically (Figure 2), and there was a diagonal stiffener in the joint zone. As stipulated by GB51022-2015 [3], the minimum thickness of SGFs plate should not be less than 3 mm, the flange width-thickness ratio should not be greater than$\;15\sqrt{235/f_{y}}$ and the web height-thickness ratio should not be greater than $250\sqrt{235/f_{y}}$. The specific design of the specimens also referred to the No. 18-6c rigid frame as illustrated in Steel Structure of Portal Frame Light House 02SG518-1 2002 [27], and the cross-sectional dimensions of the members are shown in Figure 2b. It should be noted that the design method in GB51022-2015 and 02SG518-1 2002 is based on elastic design. The depth of webs, thickness of webs, width of flanges, and thickness of flanges of rafters and columns are shown in Table 1.

**Figure 3:**
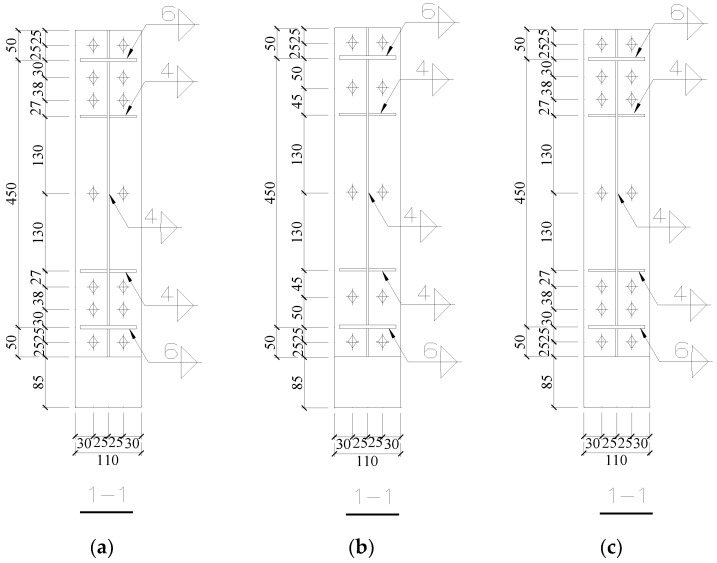
Arrangement of end-plate bolts. (**a**) SV1; (**b**) SV2; (**c**) SV3.

According to the design prototype, i.e., 18-6c Rigid frame, the specific information and details of the joint is shown in Table 2. In order to investigate the seismic behavior of the joint with reduced stiffness, the joint end-plate thickness of specimen SV1 was reduced to 8 mm and the bolt diameter was reduced to 8 mm. The bolts number of specimen SV2 was reduced to 10. The bolt diameter of specimen SV3 was reduced to 8 mm, and the steel grade was reduced to 8.8. Note that other parameters were the same as those of the design prototype. The layout of vertical end-plate bolts is shown in Figure 3.

### 2.2. Material Properties

The steel materials used in this test were all grade Q235B steel, and the properties are shown in Table 3. The bolts were friction-type high-strength bolts.

### 2.3. Test Setup and Measurement

**Figure 4:**
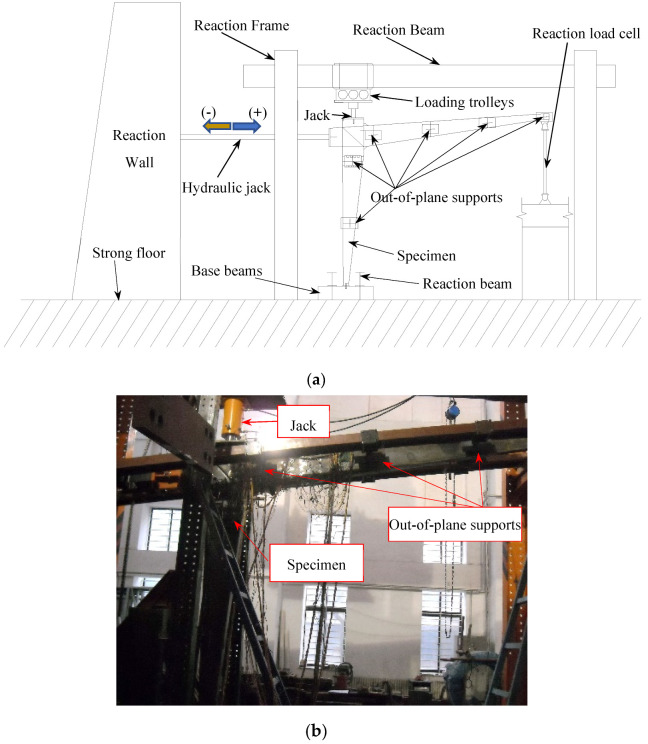
Test setup. (**a**) Diagram; (**b**) Photo.

The test setup mainly consisted of an actuator, jacking, reaction frame, reaction beams, base beams, and reaction load cell (Figure 4), in which the rafter was placed horizontally and the column placed vertically; the ground beam was connected to the strong floor. The cyclically lateral loading was applied by a hydraulic servo actuator. The axial load was applied to the column by a hydraulic jack, which was allowed to move laterally with the applied lateral loading, since a rolling guide was provided between the reaction beam and the jack. Out-of-plane supports were applied to the rafter and column at 1.5 m intervals to avoid the out-of-plane instability, which simulated and replaced purlins in the real SGF.

**Figure 5:**
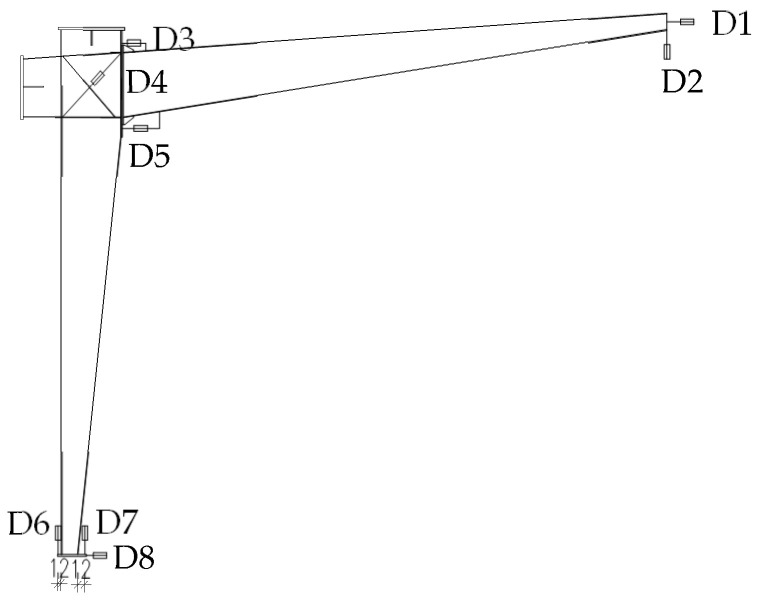
Layout of displacement gauges D1 to D8.

As shown in Figure 5, displacement gauges No. 1 (D1) and No. 2 (D2) were used to measure the lateral and vertical displacement at the tip of the rafter, respectively. Displacement gauges No. 3 (D3) and No. 5 (D5) were used to measure the lateral displacement at the top and bottom of the end-plate respectively, which were used to calculate the rotation of the end-plate. Displacement Gauge No. 4 (D4) was used to measure the relative diagonal displacement at the joint zone, which were used to calculate the joint distortion. Displacement gauges No. 6 (D6) and No. 7 (D7) were used to measure the vertical displacement at the end of the column base respectively, which were used to calculate the rotation of the column base. Displacement gauge No. 8 (D8) was used to measure the possible lateral displacement of the column base.

### 2.4. Loading Procedure

**Figure 6:**
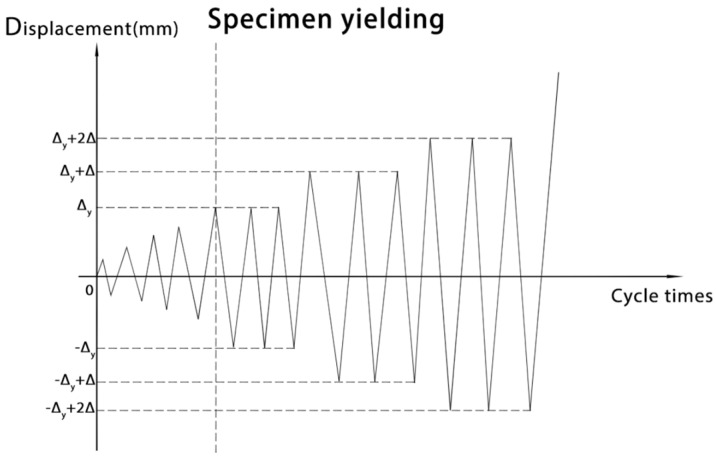
Loading procedure.

The axial load *N* applied at the top of the column was the sum of the roof load, longitudinal wall load, column self-weight, and live load (multiplied by the corresponding load factor), which was 16.14 kN. The axial load *N* was firstly applied to the top of the column at one time, and then all specimens were loaded laterally at the top of the column during testing. The load control method was used initially and graded loading was used, with each load increment of 5 kN up to 15 kN. After the load reached Δ_y_ (15 kN), the displacement control method was applied, with each displacement increment of Δ(10 mm) and cycling three times until the specimen failed. The push and pull directions of loading were referred to as positive (+) and negative (−) direction hereafter, respectively, as shown in Figure 6.

## 3. Test Observations and Failure Mode

### 3.1. Specimen SV1

**Figure 7:**
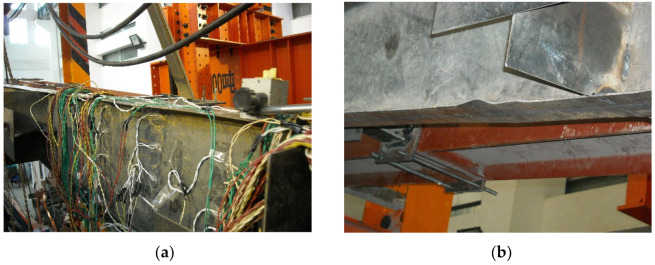
Failure phenomenon of specimen SV1. (**a**) Corrugation on the upper flange of the rafter; (**b**) Local buckling of the lower flange.

At the initial stage of loading, the rafter and column flange of specimen SV1 remained in plane. As shown in Figure 7a, the upper flange of the beam near the end-plates became corrugated when the load reached the positive maximum, and the lower flange of the rafter became corrugated when the load reached the negative maximum. As shown in Figure 7b, significant local buckling occurred at the lower flange at about 1/2 rafter span away from the end-plates, which was the final failure mode. Note that the upper flange and column showed no visible buckling, and the column base bolts were always tight and showed no signs of loosening. The stiffening rib in the joint zone did not show obvious deformation, and there was no damage in other parts of the joint in the whole testing. Since the gap between the end-plates was very small, the end-plates did not deform apparently and the bolts were not pulled off.

It should be noted that the rafter of specimen SV1 showed out-of-plane buckling at the first loading due to insufficient out-of-planes support stiffness. After straightening the rafter and strengthening the out-of-plane supports, the test was re-conducted.

### 3.2. Specimen SV2

**Figure 8:**
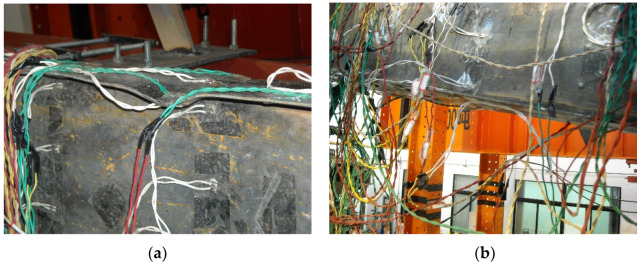
Failure phenomenon of specimen SV2. (**a**) Local buckling of the upper flange; (**b**) Local buckling of the lower flange.

As shown in Figure 8, both the upper and lower flanges of Specimen SV2 became corrugated at the positive maximum and negative maximum load, respectively; the obvious buckling of the beam upper flange and slight buckling of the lower flange occurred at 780 mm away from the end-plates. With the increase in displacement, a gap appeared between the two end-plates. The stiffening ribs and end-plates in the joint zone showed no significant deformation and no other obvious damage.

### 3.3. Specimen SV3

**Figure 9:**
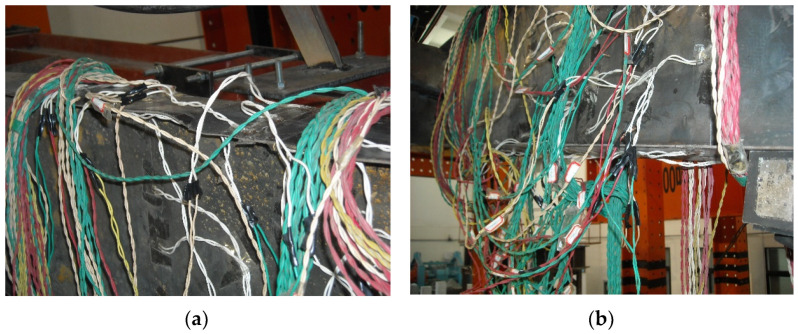
Failure phenomenon of specimen SV3. (**a**) Local buckling of the upper flange; (**b**) Local buckling of the lower flange.

As shown in Figure 9, the upper flange of the rafter of specimen SV3 significantly buckled at 680 mm from the end-plates and the lower flange slightly buckled. The final failure mode was similar to that of specimen SV2, and no damage was observed at the column and joint zone.

### 3.4. Comparison of Specimens SV1, SV2, and SV3

Specimen SV1 locally buckled at the lower flange of the rafter, which was about 1/2 beam span away from the end-plates, and then the specimen was damaged. However, both SV2 and SV3 locally buckled at the upper flange of the rafter, which was at about 1/5 rafter span away from the end-plates. All three specimens showed limited ductility as the horizontal load dropped quickly after the flange buckled. The columns of all the specimens were not damaged, meeting the seismic requirements of “strong columns and weak beams”.

## 4. Analysis of Test Results

### 4.1. Analysis of P–Δ Hysteresis Curves

**Figure 10:**
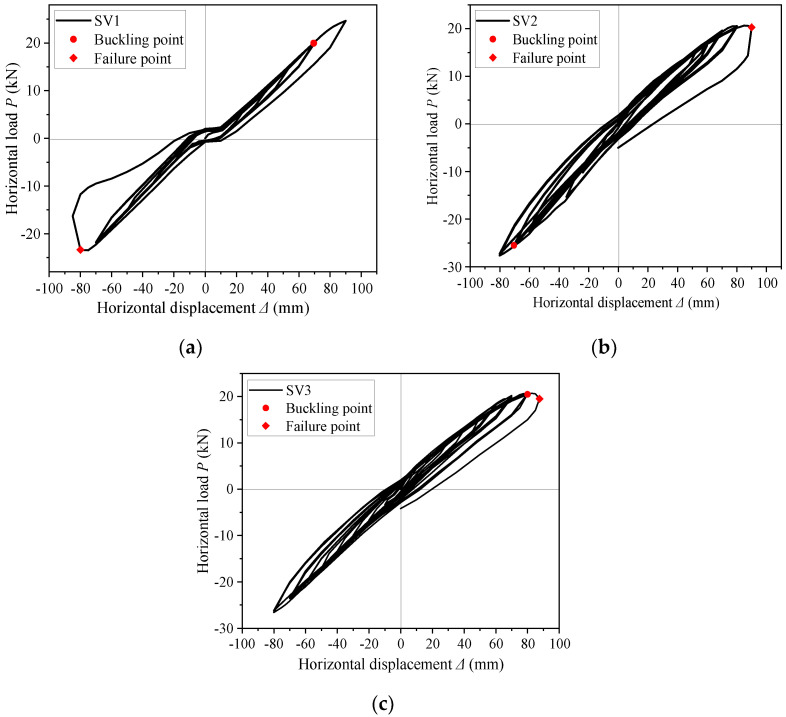
Hysteresis curves. (**a**) SV1; (**b**) SV2; (**c**) SV3.

The *P*–Δ hysteresis curves of the three specimens are shown in Figure 10. All the curves were nearly linear at the initial elastic stage. After entering the plastic stage, the shape of a single hysteresis loop was roughly shuttle-shaped, and the area enclosed by the hysteresis loop increased and the nonlinearity became obvious. The lateral displacement reached about 90 mm when the three specimens reached the peak load point in the positive direction. Then the structures buckled at the lower flange and the load no longer increased.

The analysis showed that the maximum load and the corresponding displacement of specimen SV1 were 23 kN and 80 mm under positive direction, respectively; and the maximum load and the corresponding displacement were 22 kN and 70 mm under negative direction, respectively. Thus, the difference of peak load of specimen SV1 between the positive and negative direction was very small. The peak load and the corresponding displacement of specimen SV2 were 21 N and 90 mm in the positive direction, respectively; and the peak load and the corresponding displacement were 28 kN and 80 mm in the negative direction, respectively. Thus, the peak load of the negative direction was 30% larger than that of the positive. The peak load and the corresponding displacement of specimen SV3 were 21 kN and 87 mm in the positive direction, respectively; and the peak load and the corresponding displacement were 26 kN and 80 mm in the negative direction, respectively. Thus, the peak load of the negative direction was 25% larger than that of the positive.

Hysteresis loops of specimens SV1, SV2, and SV3 exhibited significant pinching when subjected cyclic loading. The energy dissipation capacity of specimen SV3 was slightly higher than that of SV1, indicating that increasing the end-plate thickness was beneficial to energy dissipation. However, the energy dissipation of the specimen was due to the buckling located at the rafter flange rather than the end-plates, so increasing end-plate thickness hardly increased the energy dissipation capacity. For all three specimens under the same loading displacement, the shape of hysteresis loops of the previous cycle changed little when compared with the hysteresis loops of the later cycle, and the ductility and energy dissipation capacity of all the three SGFs were limited.

### 4.2. Envelope Curves

**Figure 11:**
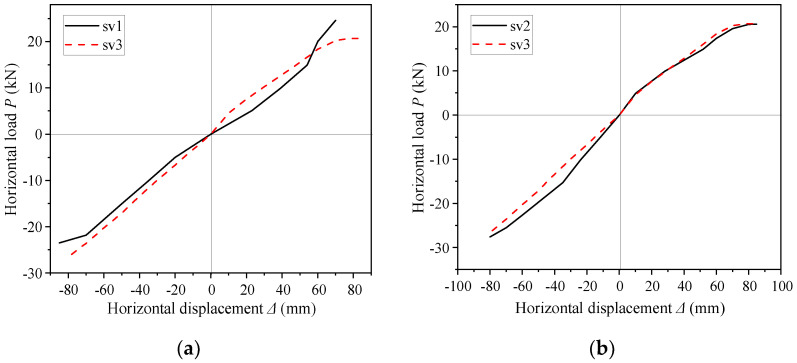
Envelope curves. (**a**) Specimen SV1 and SV3; (**b**) Specimen SV2 and SV3.

The stiffness of specimen SV3 was greater than that of specimen SV1 due to the increased end-plates thickness (see Figure 11a). However, since the final failure mode of both SV1 and SV3 was local buckling of the rafter flange but the end-plates showed no damage, the difference between the two specimens was small. The peak loads of SV2 and SV3 were almost the same (Figure 11b), so increasing the number and grade of bolts also did not affect the seismic performance of SGFs. The envelope curves of the three specimens demonstrated there was no obvious yield point and they almost kept linearly until the failure. The specimens were damaged quickly after the load reached the peak and the load–displacement curves had no obvious descending branch. The main reason for the above phenomenon was that the width-to-thickness ratio of the flange was large, i.e., the slender section was difficult to form a plastic hinge as the member buckled rapidly after yielding. Therefore, the ductility of SGFs was not good, and the energy dissipation capacity of the member after yielding was limited.

### 4.3. Comparison of Specimen Predicted, Buckling, and Peak Load Values

**Figure 12:**
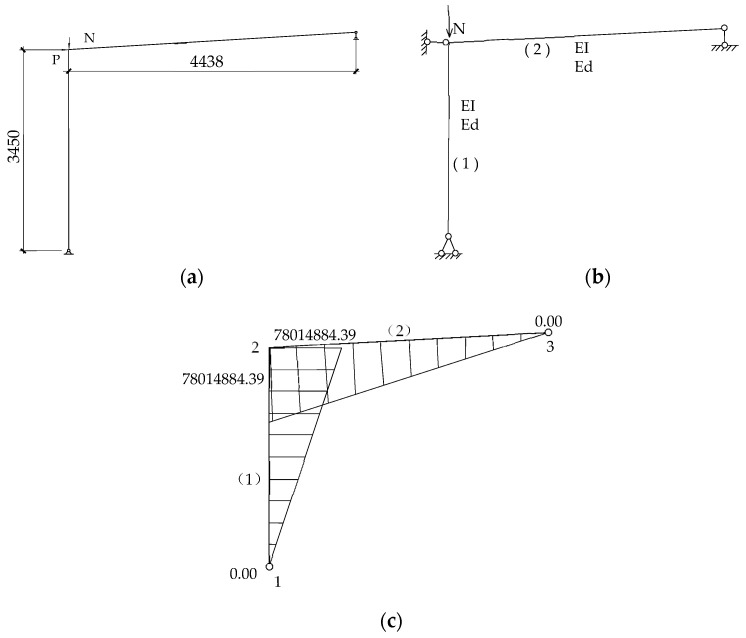
The calculation process of SGF. (**a**) Calculation sketch of the specimen; (**b**) Mechanical model for yielding load calculation; (**c**) Trial calculated internal force.

The following Figure 12 shows the calculation sketch of 18-6c rigid frame in GB51022-2015 [3]. According to the calculated sketch, the yield bending-moment near the joint was 78 kN/m, the yield displacement was 68 mm, and the corresponding yield-load under monotonic loading was 18 kN. The predicted yield load *F*_d_ and predicted yield displacement Δ_d_ of specimens SV1, SV2, and SV3 were then calculated based on the stiffness of the specimens, as shown in Table 4.

The test results including the buckling load when the rafter buckled, and the damage load when the specimen failed are shown in Table 4. The column top lateral damage load and displacement of specimen SV1 are 8.9% and 12.5% smaller than those of specimen SV3, respectively. Therefore, increasing the end-plate thickness would improve its seismic performance under the requirements of the code, but the improvement was minimal especially for the horizontal load. The peak load and displacement at the top of the column of specimen SV3 are 2.8% and 24.0% smaller than those of specimen SV2, respectively. Thus, increasing the strength of the bolts would improve the seismic performance of SGF, especially for horizontal ultimate loads.

As shown in Table 4, The lateral buckling displacement (Δ_b_) of the above three specimens increased by 0–16.5% compared with the corresponding predicted value (Δ_d_), and increased by 9.4–28.6% compared with the corresponding lateral damage displacement (Δ_u_). The lateral load at the top of the column (*F*_b_) during buckling of the three specimens was increased by 6.0–36.1% compared with the corresponding predicted value (*F*_d_), and reduced by 4.7–20.6% from the corresponding damage load (*F*_u_). Therefore, SGF rafters designed according to 02SG518-1 [27] will buckle soon after reaching the predicted load value, and will damage soon after buckling, with limited ductility and energy dissipation capacity.

For the above three specimens, the bolts were not pulled off, the end-plates were not obviously deformed or bent, and the joint webs were not damaged when specimens finally failed. This indicates that the design method of flexural capacity according to 02SG518-1 [27] about the end-plates bolted connection is more conservative and has higher safety reserve.

## 5. Finite Element Analysis

### 5.1. Finite Element Model

The plates and bolts used for the FEA model were all solid units. In order to reduce the number of elements and the solution time, this model used SOLID45 elements as much as possible. Since SOLID45 only supports prismatic hexahedral elements, the model should be divided as regular as possible. For the division of the model mesh, firstly, the model was divided into several pieces according to rafters, columns and joints, so that each piece was a six-sided prismatic body. SOLDE45 was used for “SWEEP” division of rafter-column plate parts, SOLID95 was used for “FREE” division of joint zone plates, and “FREE” division was also used for end plates and bolts.

**Figure 13:**
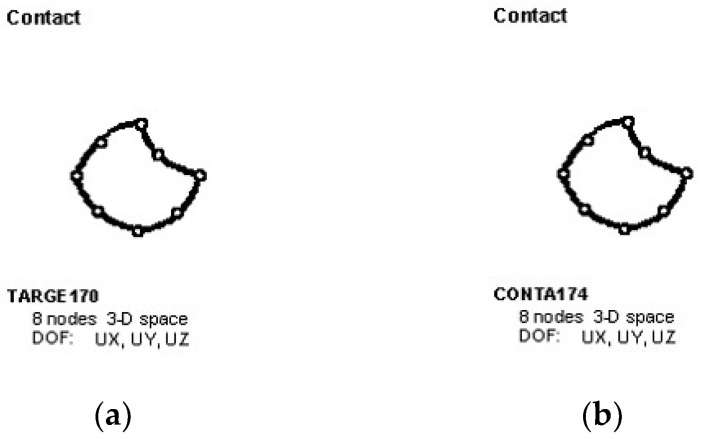
Schematic diagram of 3D contact pairs. (**a**) Diagram of TARGE170; (**b**) Diagram of CONTA174.

The contact between the two end-plates in the FEM was simulated by creating a 3D contact pair on the contact surface of the end-plates, which consists of the target unit TARGE170 (Figure 13a) and the contact unit CONTA174 (Figure 13b).

The pretension in bolts was applied by generating a 3D pretension cell PRETS179 with “PSMESH” command. Pretension cell PRETS179 is used to define a 2D or 3D pretension zone within a meshed structure that can be built from any 2D or 3D structural cell. This cell can be used only for tensile loads, ignoring bending or torsional loads. The pretension zone consisted of a set of PRETS179 cells, which could easily apply load or displacement to the pretension zone. The PRETS179 cells used one translational degree of freedom to represent the relative displacement in the pretension zone along the pretension direction. The pretension load was first applied to the pretension zone, and then the displacement of the pretension zone was fixed while the working load was applied, to simulate the pretension load application and the normal working condition of high-strength bolts.

Based on the experimental study, a refined finite element model using ANSYS software was established. The model considered the large deformation of the specimen, the surface-to-surface non-linear contact of the high-strength bolted connection, and the plasticity of the steel to simulate the cyclic performance of the test specimens.

The geometric model was established by entering the coordinates of the key points. The end-plate contact surfaces had no initial geometric clearance. The bolt was modeled without considering the threads, and nuts were modeled approximately using a circle shape. The bolt diameter was taken as the nominal diameter since the cross section might be reduced due to meshing.

The adopted stress–strain curve of Q235B steel is shown in Figure 11a, and the stress–strain curve of high-strength bolts is shown in Figure 11b.

**Figure 14:**
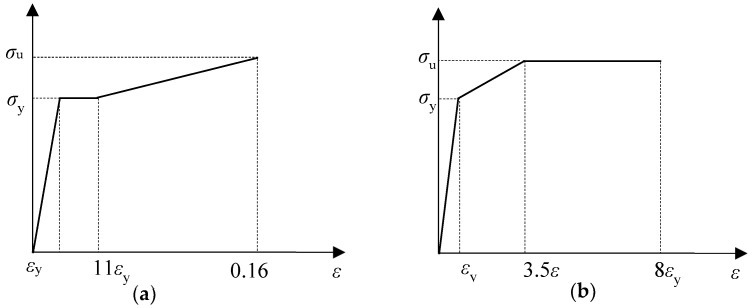
Steel principal structure curves. (**a**) Steel Q235B; (**b**) High-strength bolts.

In Figure 14, *σ*_y_ is the yield stress of the steel tested and *σ*_u_ is the maximum stress of the steel tested, the values of which are taken from Table 3. The corresponding strain *ε*_y_ is the corresponding yield strain, which is equal to the yield stress divided by the modulus of elasticity.

**Figure 15:**
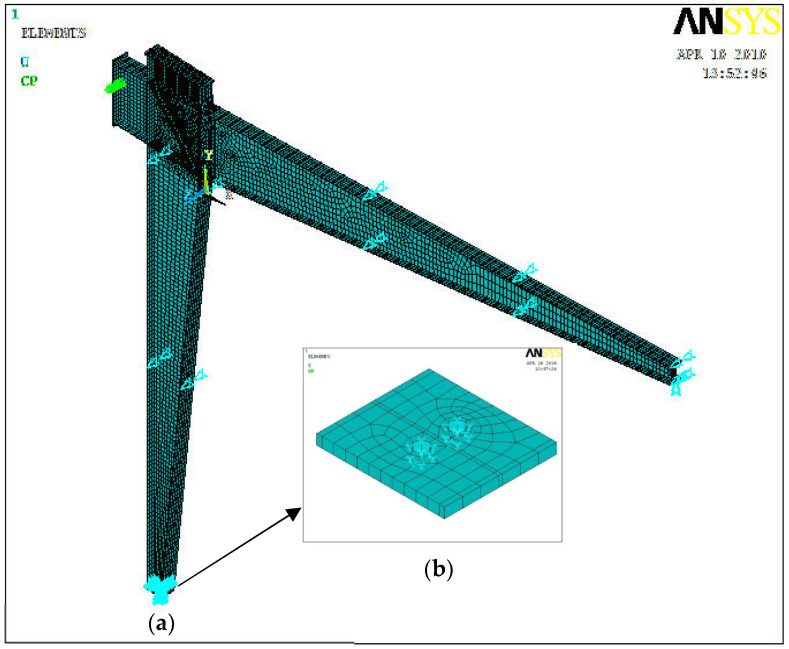
ANSYS model. (**a**) SGFs model; (**b**) Model of column base.

Both the column base and beam end of the specimen model were hinged (Figure 15a). In the analysis, the axial load *N* was applied firstly at the column top, and then the cyclically lateral load was applied at the top of the column. The out-of-plane restraints were applied to the beam and column at 1.5 m intervals, aiming to prevent the unexpected out-of-plane instability failure mode, which was used to model the braces in SGFs. The bolted connection of the column base was simulated, holes were drilled in the base plate of the column, and the boundary constraints were applied around the holes, as shown in Figure 15b.

The axial load of 16.4 kN was applied to the top of the column by a surface loading method. The bolts were pre-tensioned. Then, cyclically lateral displacement was applied at a coupling point on the end surface of the rafter, by which a set of nodes were allowed to move in the translational direction. The lateral load was first applied monotonically, and then was applied cyclically twice.

### 5.2. Finite Element Analysis Results

**Figure 16:**
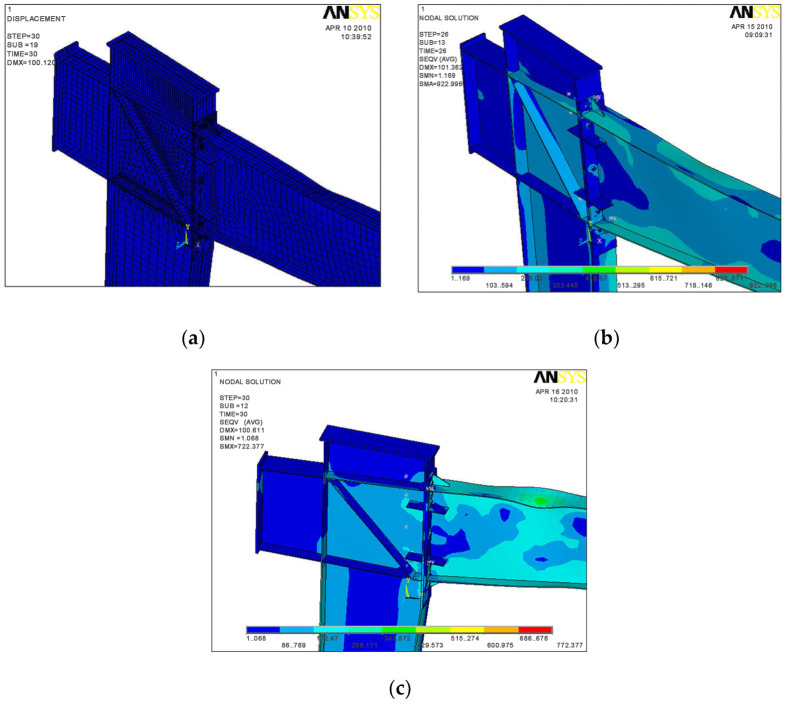
FEA model failure mode. (**a**) SV1; (**b**) SV2; (**c**) SV3.

The failure models of the three specimens are shown in Figure 16. When model SV1 was loaded to 90 mm, the upper flange buckled at 620 mm away from the end-plate, and the lower flange buckled at 590 mm away from the end-plate. When model SV2 was loaded to 90 mm, the upper flange buckled at 689 mm away from the end plate, and l the lower flange buckled at 656 mm away from the end-plate. When model SV3 was loaded to 90 mm, the upper flange occurred buckled at 500 mm away from the end-plate, and the lower flange buckled at 586 mm away from the end-plate. The failure locations of all the three specimens were roughly 1.3–1.5 times the height of the beam away from the end-plate, accompanied by local buckling of the web. Therefore, the FEA model effectively predicted the test observations, failure modes, and failure locations.

**Figure 17:**
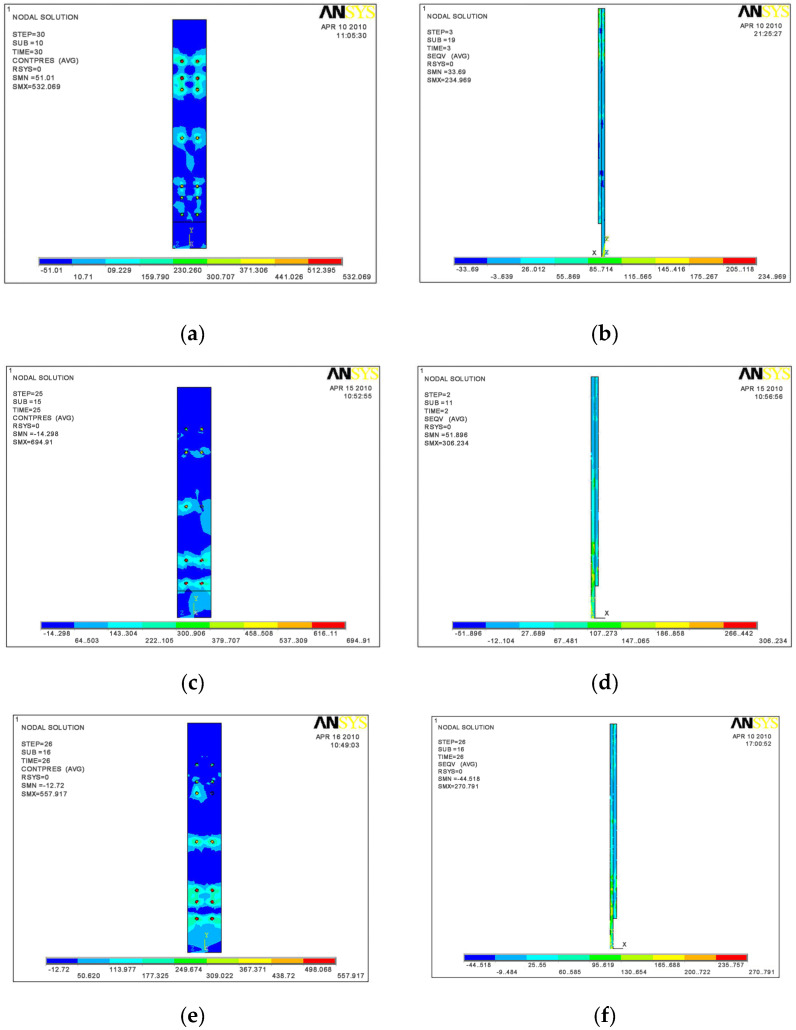
End-plate stress diagrams when specimen failed. (**a**) Contact surface stress of specimen SV1; (**b**) Stress of specimen SV1; (**c**) The contact surface stress of specimen SV2; (**d**) Stress of specimen SV2; (**e**) Contact surface stress of specimen SV3; (**f**) Stress of specimen SV3.

Figure 17 shows the stress distribution of the end-plate when the specimens failed. The stress of the end-plate was less than 180 MPa except around the bolt holes, which agreed with the test observations that the end-plate had small deformation.

**Figure 18:**
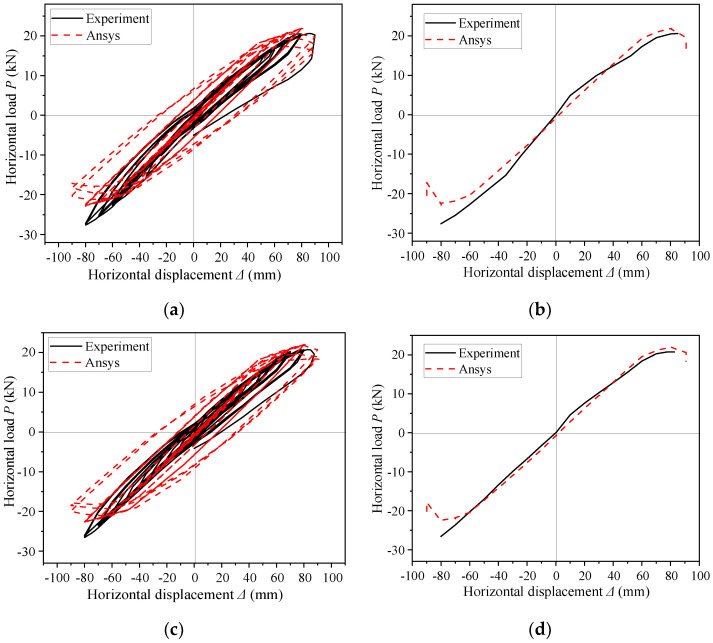
Comparison of numerical analysis results with test results. (**a**) *P*–Δ curve of specimen SV2; (**b**) Envelope curves of specimen SV2; (**c**) *P*–Δ curve of specimen SV3; (**d**) Envelope curves of specimen SV3.

The P–Δ curves and envelope curves of specimens SV2 and SV3 are shown in Figure 18. Since specimen SV1 showed out-of-plane instability during the first-round loading and was modified for a second-round loading, its test load–displacement curves were not used for comparison with the FEA results. Generally, the numerical simulation showed satisfactory agreement with the test, and the numerical model showed a little smaller load at 80–90 mm in the negative direction when compared with the test results. The reasons might be (1) the steel strengthening section was not considered in the ANSYS numerical simulation, while the steel in the test had a strengthening section; (2) the actual value of bolt pre-tension differed from the design value due to the unevenness of the end-plate, while the design value was applied exactly in the finite element model.

**Figure 19:**
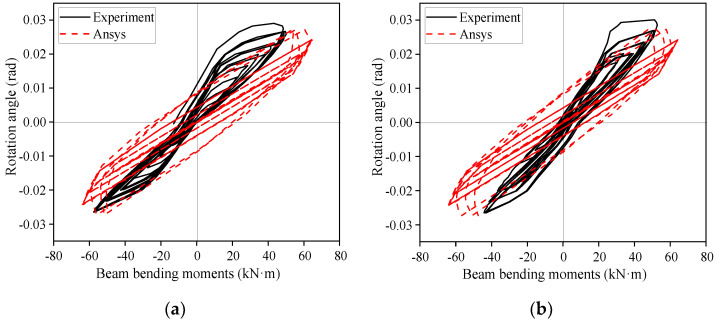
Comparison of joint bending moment–rotation curves. (**a**) SV2; (**b**) SV3.

The moment–rotation curves of specimens SV2 and SV3 are shown in Figure 19, in which Mc is the bending moment of the joint (Mc = RL, where R is the reaction force at the beam end and L is the span of the rafter); θ is the angle of rotation of the rafter–column joint (θ = Δ/H, where Δ is the lateral loading displacement, H is the distance from the loading point to the column base). Generally, the predicted results were acceptable. As can be seen from Figure 19, the stiffness of the numerical model is larger than that of the test, but θ are close, mostly because the beams of the same rafter span L but different R in the numerical model and the test. In the test, the rafter end (Figure 4) was not fully re-strained from vertical displacement due to its ability to oscillate in the horizontal direction to a small degree. In the numerical model, the vertical displacement of the rafter end was fully restrained (Figure 15).

### 5.3. Discussion and Design Recommendation

From the test results and FEA results, even though the flexural capacity and stiffness of the joint were weakened on the basis of the prototype 02SG518-1 specimen, no damage occurred in the whole joint zone during the test. Therefore, the joint zone of the prototype SGF designed in accordance with the design guideline 02SG518-1 has adequate margin of safety. In addition, both results of test and FEA showed that the specimen were damaged quickly after buckling, so it is reasonable that the post-buckling strength of the non-compact and slender webs should not be considered in seismic design.

Therefore, the seismic performance of SGF mainly depends on its own load-carrying capacity, rather than resisting the seismic force through energy dissipation or ductile deformation. Thus, the seismic design of SGFs joint zone only needs to be designed according to GB51022-2015. The joint zone designed according to GB51022-2015 has sufficient safety reserves.

## 6. Conclusions

The seismic behavior of three 1/2-scale, half-bay SGFs was experimentally investigated. The effects of the end-plates thickness, bolt diameter, and bolt arrangement on the seismic performance of specimens were analyzed, and a refined finite element model was established. The following conclusions can be drawn:

(1) The test results demonstrated all the three SGF specimens satisfied the “strong column and weak rafter” design. The three specimens all failed due to local buckling of the rafter flange, while the column and joint region were almost intact. The location of the plastic hinge was at a distance about 1/5 or 1/2 of the rafter span from the end-plates, indicating that the location of the plastic hinge was different from that of a commonly used steel frame structure.
(2) The hysteresis curves of all the specimens were not full, the ductility was not favorable, and the post-buckling energy dissipation capacity of the members was limited. The capacity of the specimen decreased quickly once the web buckled. Therefore, the seismic performance of SGF mainly depends on its own bearing capacity, rather than resisting the seismic force through energy dissipation or ductile deformation.
(3) Since no failure occurred at joints, varying the thickness of the end-plate, the strength and number of the bolts could only slightly affect the ductility and capacity of SGFs. The finite element analysis results compared well with the test results, further illustrating the minor effects of the test variables on the seismic performance. Consequently, the flexural capacity design of the bolted end-plate connection illustrated in the design guideline 02SG518-1 is on the safe side. Therefore, the seismic design of SGFs joint zone could only been designed according to GB51022-2015.
(4) There was slight post-buckling strength of the web as shown in the experimental and finite element analysis results. The web always carried the axial stresses generated by the bending moment, and there was slight utilization and distribution of post-buckling strength.

## Figures and Tables

**Table 1:** Specimen dimensions.

Members	Depth of Webs (mm)	Thickness of Webs (mm)	Width of Flanges (mm)	Thickness of Flanges (mm)
Rafters	110–444	3	100	4
Columns	115–436	3	110	5

**Table 2:** Specimen parameters.

Specimen	Thickness of End-Plate	Diameter of Bolts	Grade of Bolts	Number of Bolts
18-6c Rigid frame [27]	10	10	10.9	14
SV1	8	8	8.8	14
SV2	10	10	10.9	10
SV3	10	8	8.8	14

**Table 3:** Material testing properties.

Components	Thickness (Diameter) (mm)	Tensile Strength (MPa)	Yield Strength (Mpa)	Elastic Modulus (×10^5^N/mm^2^)	Elongation (%)	Yield Strain (%)
Plates	2.703	443	309	2.043	35.027	0.155
	3.567	497	363	2.107	34.053	0.179
	4.557	491	352	2.063	31.720	0.175
	7.357	438	284	2.233	42.573	0.167
	9.343	462	293	1.930	42.280	0.153
Bolts	8	830	504	-	-	0.245
	10	1040	632	-	-	0.307

**Table 4:** Test results.

	Specimen		
	SV1	SV2	SV3
Predicted load *F*_d_ when rafter yielding (kN)	16.846	18.740	20.391
Buckling load *F*_b_ (kN)	19.960	−25.500	20.506
Damage load *F*_u_ (kN)	−23.370	20.259	19.540
Peak load *F*_p_^(+)^	24.720	20.814	19.540
Peak load *F*_p_^(−)^	−23.370	−27.774	−26.57
*|F*_b_/*F*_d_|	1.185	1.361	1.006
*|F*_u_/*F*_b_*|*	1.171	0.794	0.953
*|F*_u_/*F_d_|*	1.387	1.081	1.333
Predicted displacement when rafter yielding Δ_d_ (mm)	59.995	69.973	74.989
Buckling displacement Δ_b_ (mm)	69.900	−70.000	79.982
Damage displacement Δ_u_ (mm)	−80.000	89.997	87.500
Δ_b_/Δ_d_	1.165	−1.144	−1.333
Δ_u_/Δ_b_	−1.000	−1.286	1.286
Δ_u_/Δ_d_	1.067	1.094	1.167

The predicted load *F*_d_ is the lateral load when the rafer yielded, and Δ_d_ is the corresponding displacement.

## Data Availability

The data that support the findings of this study are available from the corresponding author upon reasonable request.
